# Anisotropic transport induced by DC electrical current bias near the critical current

**DOI:** 10.1038/s41598-020-73876-6

**Published:** 2020-10-08

**Authors:** Junghyun Shin, Sungyu Park, Eunseong Kim

**Affiliations:** 1 Department of Physics, KAIST, Daejeon, 34141 Republic of Korea; 2 Present Address: Center for Artificial Low Dimensional Electronic Systems, Institute for Basic Science (IBS), Pohang, 37673 Republic of Korea

**Keywords:** Mathematics and computing, Nanoscience and technology, Physics

## Abstract

We investigated the transport characteristics of a square shape superconducting Ta thin film under DC electrical current bias along the diagonal direction. The resistance parallel (*R*_*∥*_) and perpendicular (*R*_*⊥*_) to the DC current, *I*_*DC*_, is measured with various magnetic fields. *R*_*∥*_ and *R*_*⊥*_ show contrasting dependence on *I*_*DC*_. First, the critical current of *R*_*∥*_ is smaller than that of *R*_*⊥*_. Second, *R*_*⊥*_ shows an unexpected reduction at current bias where *R*_*∥*_ shows a rapid increase near the transition from a flux flow state to a normal state. The intriguing anisotropic transport characteristics can be understood by the inhomogeneous current density profile over the square sample. Diagonal DC current induces an anisotropic current density profile where the current density is high near the biasing electrode and low at the center of the sample. Accordingly, the electrical transport in the perpendicular direction could remain less affected even near the critical current of *R*_*∥*_, which leads to the higher critical current in *R*_*⊥*_. Complicated conduction profile may also allow the anomalous reduction in the *R*_*⊥*_ before finally shifting to the normal state.

## Introduction

The vortex dynamics in superconducting thin films near the phase transitions has been intensively studied with the current–voltage (IV) characteristics^1–4^ and various microscopies such as scanning superconducting quantum interference device microscopy^5^, Hall probe microscopy^6^, scanning laser microscopy^7^, and scanning tunneling microscopy^8^. In addition, recent understanding of vortex dynamics has been improved through the simulation of the time-dependent Ginzburg–Landau equation^9^. In superconducting thin films, when a magnetic field between *B*_*C1*_ where Meissner state disappears and *B*_*C2*_ which shows the transition to the normal state is applied, quantized vortices characterized with a quantum flux of *Φ*_*0*_ enter the superconducting films. These vortices experience various interactions such as vortex-vortex repulsion, localization by pinning centers, and the Lorentz force driven by the electrical current. Distinctive transition in the vortex motion can be achieved by gradually increasing current. The pinned vortex state appears in the low current region. The flux flow state can be found when the Lorentz force exceeds the pinning in the intermediate range of current. The normal state is obtained when exceeding the critical current. Thus, the transition in the vortex motion can be investigated by measuring the IV characteristics.

The superconducting to normal transition in the IV curves is identified by a sudden jump or non-linearity in the voltage response near the critical current^1–4,10–12^. The transition can be understood by a vortex instability provided when the vortex velocity driven by the Lorentz force exceeds the critical vortex velocity^1,2,10^. On the other hand, the transition may not be solely caused by the electric current because a similar transition can be induced by hot electrons at low temperatures^2,3,11^. Although the vortex instability at low temperatures is less significant, essentially the same transition can be obtained and understood within the hot electrons framework. Therefore, both effects are relevant in destructing two dimensional (2D) superconductivity.

In general, the superconductor-insulator transition at the zero-temperature limit has been systematically studied by controlling the thickness and magnetic field^13–17^. Thus, it is intriguing to study the effect of a strong DC electrical current bias on the transition at various temperatures and magnetic fields. For instance, suppose a strong electrical current is applied to a superconducting thin film. An anisotropic vortex structure may be constructed near the critical current^5–7,9^, which can be detected with the discrepancies between the horizontal and vertical transport to the direction of the current. Here, we fabricated superconducting Ta thin films in square geometry and studied the transport characteristics while a DC electrical current was applied between two facing vertices. *R*_*∥*_ and *R*_*⊥*_ were mainly investigated near the critical current. The unexpected reduction in *R*_*⊥*_ and contrasting critical current values between *R*_*∥*_ and *R*_*⊥*_ were observed near the transition and discussed in relation to the current density profile in the square geometry and vortex dynamics.

## Results and discussion

**Figure 1:**
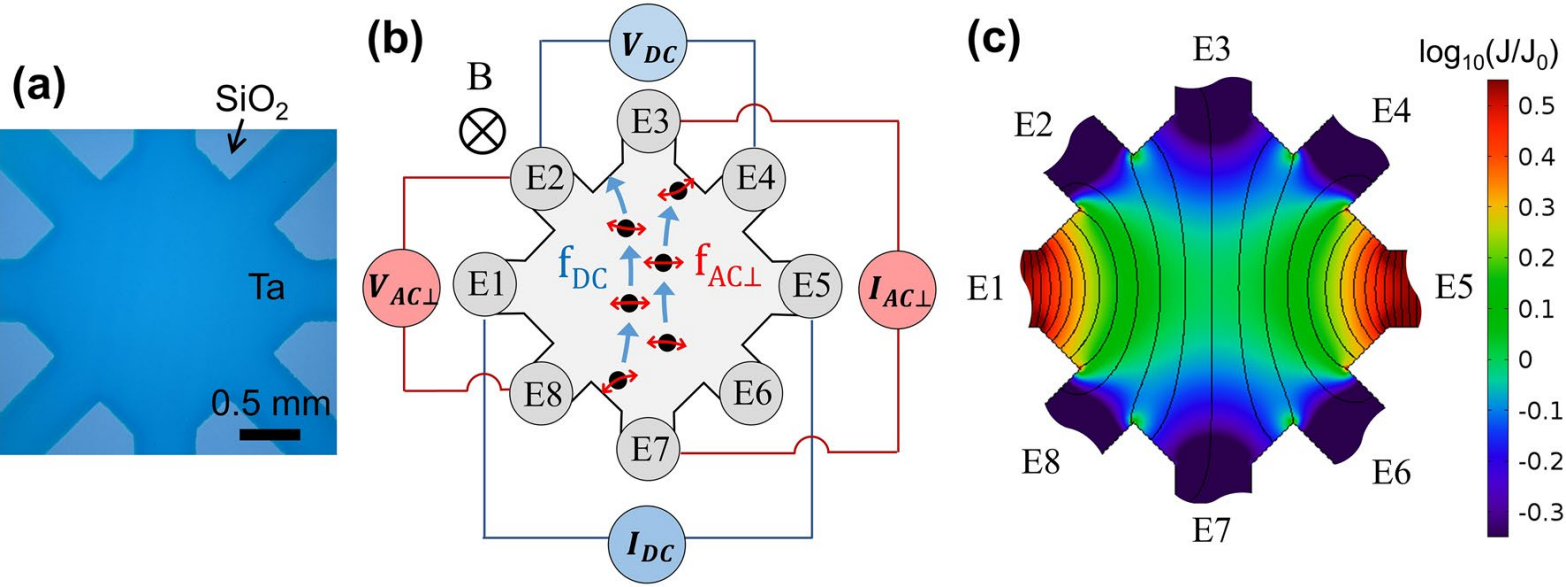
Transport measurement and simulation in a square sample. (**a**) Microscopic image of a real device in a square geometry. (**b**) Schematic diagram of measurement configuration and vortex movements in the square sample geometry. Each electrode is numbered such as E1-E8. Magnetic field is applied perpendicular to the sample surface. The black circles illustrate quantized vortices. The blue arrow (red arrow) represents the direction of ***f***_***DC***_ (***f***_***AC⊥***_), Lorentz force per single vortex arising from DC bias current (AC current applied perpendicular to DC bias current). (**c**) Simulation result of the current density profile in square sample with a constant conductance, which is an equivalent result of the normal metal. The DC bias current is applied along the diagonal direction between E1 and E5. The colored contour map represents the logarithmic current density (*J*) normalized to the value(*J*_*0*_) at the center of the sample. The black solid line shows equipotential lines induced by the DC bias current. The simulation image is generated with COMSOL Multiphysics 5.0 (https://comsol.com).

Ta thin films with various thicknesses were prepared for anisotropy measurements. The fabricated Ta films show homogeneous and amorphous characteristics^18^. The sample shape with the 8 electrodes configuration is shown in Fig. 1a. The sample was fabricated in square geometry with 2 mm length on one side. Wide electrodes with 0.5 mm width were placed at the center of all vertices and the middle points of each side to measure electrical conductivity. While the sample shape and electrode configuration are similar to those of the van der Pauw method^19,20^, the measurement configurations and the physics intended to study are different. The van der Pauw technique needs only four probes at the corners and conducts series of resistance measurements in various geometrically identical current voltage configurations. An average resistivity of a thin film with homogeneous thickness is, then, determined by the van der Pauw formula. Instead, we use 8 electrodes at four corners and four mid-points of sides to measure the transport properties parallel to and perpendicular to the DC current bias simultaneously.

For a clear distinction, the electrodes are numbered from E1 to E8 as depicted in Fig. 1b. When a DC current was applied between E1 (I+) and E5 (I-) terminals, an inhomogeneous current density distribution on the sample appears as shown in Fig. 1c. The current density profile on the sample geometry is calculated by a finite element method (FEM) simulation based on the assumption that the system shows an ohmic behavior which is marked by the fact that the conductance is constant in the sample regardless of a DC current. (The details are described in the "Methods" section.) For clarity, the current density is normalized by the value at the center of the sample and plotted in a logarithmic scale. It is notable that the current density has the maximum near E1 and E5 and the minimum at the diagonal connecting E3 and E7 where the widest current path is present. The anisotropic current density profile differs from the previous transport characteristics studies with an inhomogeneous current density^21–23^. In a steady state, this current density distribution, if normalized, is basically determined by system geometry regardless of the applied current and conductance of a system. This simulation is only suitable to describe the current density profile of a normal metal and steady-state superconductor which shows a clear discrepancy with that of a non-steady-state superconductor near the transition. We will discuss this simulation later.

We used both DC bias in the horizontal direction between E1 and E5 and AC bias in the vertical direction between E3 and E7 for measuring the transport characteristics in both directions simultaneously, which was previously adopted to identify the directional dependence of a vortex pinning force^24^. Sinusoidal AC current bias was applied with a driving frequency of 13.55 Hz and amplitude of 100 nA which is two orders of magnitude smaller than the critical current. While AC bias with a magnitude close to the critical current and driving frequency of a few kHz revealed the intriguing vortex dynamics^25,26^, extremely small AC bias at the low frequency limit is insignificant to unpin vortices. Besides, the vortex dynamics becomes important in the weakly localized vortex regime induced by strong DC bias where superimposed transverse AC bias may play a significant role in depinning the vortices^24^. However, the magnitude of the used *I*_*AC*_ is two orders of magnitude smaller than that of the critical current and, thus, too small to induce any critical change in the IV characteristics.

The schematic measurement configuration and vortex movement are shown in Fig. 1b. We measured three different types of resistance of *R*_*DC*_, *R*_*AC∥*_, and *R*_*AC⊥*_ to analyze the transport characteristics of Ta films. First, the DC resistance, $R_{DC} = V_{DC} /I_{DC}$, is obtained by measuring the DC voltage, *V*_*DC*_ (E2-E4), generated by the DC current, *I*_*DC*_ (E1-E5). The vortices injected by the perpendicular magnetic field are subjected to the Lorenz force, ${\varvec{f_{DC}}} = {\varvec{J_{DC}}} \times {\varvec{\Phi_{0}}}$ where ***f***_***DC***_ (blue arrows in Fig. 1b) is the force per a single vortex, ***J***_***DC***_ is the DC current density, and ***Φ***_***0***_ is the fluxoid quantum. The vortices are driven in the vertical direction against *I*_*DC*_. Second, we measured the parallel AC voltage, *V*_*AC∥*_, between E2 and E4 by superimposing the sinusoidal AC current, *I*_*AC∥*_(= 100 nA) with 7.77 Hz, on *I*_*DC*_ to obtain the parallel differential resistance, $R_{AC\parallel } = V_{AC\parallel } /I_{AC\parallel }$(not displayed in Fig. 1b for clarity). Third, the sinusoidal AC current, *I*_*AC⊥*_(= 100 nA) with 13.55 Hz, perpendicular to *I*_*DC*_ is applied between E3 and E7 and the perpendicular AC voltage, *V*_*AC⊥*_, is measured between E2 and E8 to obtain the perpendicular differential resistance, $R_{AC \bot } = V_{AC \bot } /I_{AC \bot }$. When *I*_*AC⊥*_ is applied between E3 and E7, Lorenz force, ***f***_***AC⊥***_(red arrows in Fig. 1b), due to *I*_*AC⊥*_ is added. We compared the three measured resistances obtained at various temperatures. We can confirm the isotropic transport characteristics when *I*_*DC*_ is identical to *I*_*AC⊥*_. *R*_*DC*_ and *R*_*AC⊥*_ at *I*_*DC*_ and *I *_*AC⊥*_ = 100 nA overlap in various temperatures and magnetic fields with the minor discrepancy which can be attributed to the misalignment of the electrodes. During the resistance measurements, the voltage decrease was measured between voltage probes where the current density varied continuously. Thus, the voltage decrease is not uniform between the probes in contrast to uniform profile in a standard four point probes measurement. One may notice this from the fact that the spacing between the equipotential lines (see solid lines in Fig. 1c) varies along the diagonal between E1 and E5. The resistance of *R*_*DC*_, *R*_*AC∥*_, and *R*_*AC⊥*_ can be estimated from the ratio of the voltage decrease between the voltage probes to the applied current when the conductance remains constant. However, the resistance is not exactly the same as, but proportional to, the real resistance between the voltage probes if the conductance varies depending on the magnitude of the current density. (We will discuss this non-steady state condition below.) Even though the estimated ratio does not give the exact resistance at this special condition, it was necessary to estimate the resistance in this practical way to describe the transport properties of the square Ta films because the direct measurement of the current density profile is impossible.

The data presented in the main text are obtained from the Ta film of a thickness of 4 nm. (DC bias dependent transport properties in Ta films with different thickness of 3.4 and 3.7 nm are included in the Supplementary Information.) The normal resistance at *T* = 1 K of the sample is 738 Ω with *T*_*C*_ = 400 mK and *B*_*C*_ = 0.68 T. The critical current where the resistance value changes abruptly to the normal state value is about 17.5 μA. The estimated critical current density is 2.13 MA/m^2^ and the details of the estimation method are discussed in the "Methods" section.

**Figure 2:**
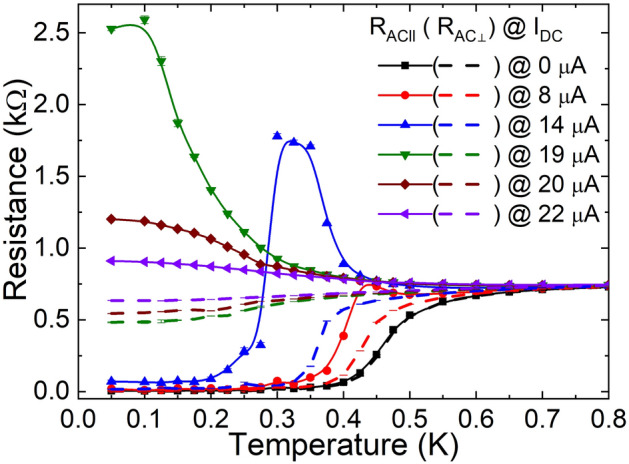
Simultaneous measurement of *R*_*AC∥*_ and *R*_*AC⊥*_. *R*_*AC∥*_ and *R*_*AC⊥*_ under various *I*_*DC*_ biases and zero magnetic field are plotted as a function of temperature. Solid symbols with straight lines are differential resistance *R*_*AC∥*_ and empty symbols with dashed lines are differential resistance *R*_*AC⊥*_. The data points are raw data and the curves are guides to the eyes.

The temperature dependence of *R*_*AC∥*_ and *R*_*AC⊥*_ was simultaneously measured under various *I*_*DC*_ biases as shown in Fig. 2. *R*_*AC∥*_ and *R*_*AC⊥*_ show the same temperature dependence as anticipated in a homogenous thin film when *I*_*DC*_ = 0. On the other hand, *R*_*AC⊥*_ shows a clear departure from *R*_*AC∥*_ near the onset of superconductivity when *I*_*DC*_ is finite. The separation between *R*_*AC∥*_ and *R*_*AC⊥*_ becomes more pronounced when *I*_*DC*_ increases. *R*_*AC∥*_ shows a non-monotonic temperature dependence indicating that the change of $V_{DC} /I_{DC}$ is sensitive to the measurement temperature. For instance, with *I*_*DC*_ = 14 μA, *R*_*AC∥*_ exhibits negative $dR_{AC\parallel } /dT$ slope above a certain temperature, *T*_*P*_, until converging to the normal state resistance at high temperature limit. Below *T*_*P*_, *R*_*AC∥*_ decreases rapidly due to the onset of superconductivity. As *I*_*DC*_ increases the peak temperature, *T*_*P*_, tends to decrease and is eventually pushed to very low temperature, which reflects an abrupt onset of a normal state at low temperatures. When *I*_*DC*_ becomes larger than 19 μA, *T*_*P*_ below which *R*_*AC∥*_ decreases disappears, indicating that *T*_*P*_ is reduced below the base temperature of our measurement. It is notable that the negative $dR_{AC\parallel } /dT$ slope decreases with increasing *I*_*DC*_. However, *R*_*AC⊥*_ shows monotonic temperature dependence which exhibits a gradual increase until saturating to the normal state resistance. The onset temperature of finite resistance is gradually lowered with broadening the transition as *I*_*DC*_ increases. With *I*_*DC*_ greater than 19 μA, *R*_*AC⊥*_ remains finite even at the temperature less than 50 mK and shows a positive $dR_{AC \bot } /dT$ slope. The clear contrast can be attributed to not only the influence of the DC current but also the thermal fluctuation at finite temperatures. Vastly populated thermal vortices at high temperatures make the transition to be more dynamic and the separation of the true DC current effect becomes intangible. To focus on the effect of DC current on the transport only, we investigated the electronic transport of the IV curves at the low temperature.

**Figure 3:**
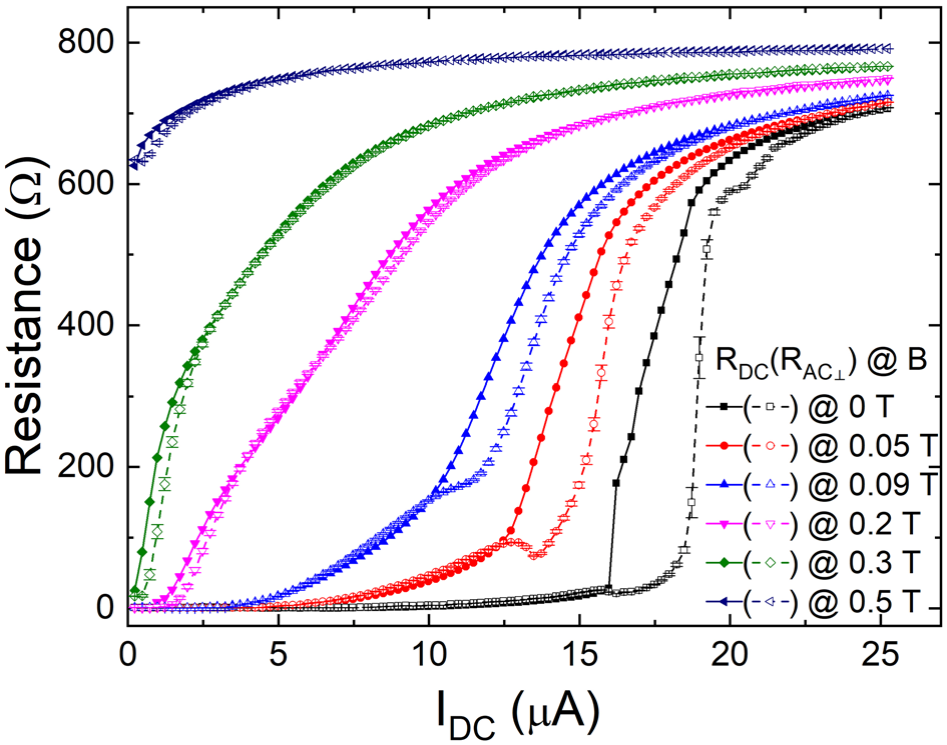
Simultaneous measurement of *R*_*DC*_ and *R*_*AC⊥*_. *I*_*DC*_ dependence of *R*_*DC*_ (solid symbols with straight lines) and *R*_*AC⊥*_ (open symbols with dashed lines) perpendicular to *I*_*DC*_ with various magnetic fields. The data points are raw data and the curves are guides to the eyes.

Figure 3 shows *R*_*DC*_ and *R*_*AC⊥*_ for various magnetic fields as a function of *I*_*DC*_ at 20 mK. When the magnetic field is not applied, *R*_*DC*_ and *R*_*AC⊥*_ at low *I*_*DC*_ region show very similar *I*_*DC*_ dependence as anticipated to be identical in homogeneous superconducting thin films. However, near the critical current region, *R*_*DC*_ and *R*_*AC⊥*_ show substantially contrasting *I*_*DC*_ dependence even at the zero magnetic field. With *I*_*DC*_ greater than 16 μA, *R*_*DC*_ exhibits a sudden increase without a change in *R*_*AC⊥*_. Rapidly increasing *R*_*DC*_ saturates finally to the normal state resistance value with a further increase of *I*_*DC*_. The deviation of *R*_*DC*_ from its low resistance state was first seen at 16 μA and increased rapidly up to 18.75 μA. *R*_*DC*_ near the critical current, *I*_*C*_*′*, does not increase abruptly in contrast to the reported IV characteristics of hall-bar shape Ta films^12^. With a sufficiently high magnetic field, non-linear IV curves with a rather gradual change of *V*_*DC*_ near the transition were found in the hall-bar samples and associated the appearance of the quantum metallic state. In addition, the signature of the direct superconductor to normal state transition was captured with a sharp resistance jump at low temperatures and with very low magnetic fields. However, the gradual onset observed in our IV measurement can be the consequence of the spatial distribution of the current density profile in a square Ta sample as illustrated in Fig. 1c. For instance, the superconducting state remains at the center of the sample while the superconducting state near the electrode E1 and E5 is destroyed. Thus, the gradual onset of *R*_*DC*_ indicates the progressive extension of the normal state resistance with increasing *I*_*DC*_. The sharp onset in *R*_*AC⊥*_ at the critical current, *I*_*C*_, on the other hand, is associated with the fact that the superconducting state in the current path of *I*_*AC⊥*_ is destroyed suddenly with increasing *I*_*DC*_. Besides, the spatially anisotropic current density distribution explains the difference in the onset currents between *R*_*DC*_ and *R*_*AC⊥*_. *R*_*AC⊥*_ increases at the higher critical value of 18.5 μA than that of *R*_*DC*_ by about 2.5 μA at the critical values where two curves vary most rapidly with increasing *I*_*DC*_.

The addition of vortices with a non-zero magnetic field induces notable changes in the IV characteristics. First, non-zero magnetic field enhances the positive slope in the IV curve in the low *I*_*DC*_ region where the resistance increases progressively. For instance, both resistances, *R*_*DC*_ and *R*_*AC⊥*_, at *B* = 0.05 T increase more rapidly than those with *B* = 0 T. It can be attributed to the fact that the vortex dynamics in the superconducting films becomes more important because of the increasing vortex population subject to the Lorentz force under finite *I*_*DC*_. The discrepancy between *R*_*DC*_ and *R*_*AC⊥*_ becomes notable with increasing magnetic field. *R*_*AC⊥*_ shows a smaller value than *R*_*DC*_ at the magnetic fields higher than 0.2 T, indicating that the resistance due to the vortex motion becomes more susceptible in the perpendicular direction with increasing *I*_*DC*_. Second, the onset current of the sudden resistance jump is reduced with increasing magnetic fields without altering the characteristic difference between the onset currents. The suppression of *I*_*C*_*′* (or *I*_*C*_) with increasing magnetic field can be understood with the simple weakening of superconductivity. In addition, the ratio of *I*_*C*_*′* / *I*_*C*_ remains essentially the same despite increasing magnetic field, demonstrating that the difference is mainly originated from the current density profile in the sample. In addition to the suppression, unanticipated reduction of *R*_*AC⊥*_ is observed at a certain *I*_*D*_. It is most pronounced at the magnetic field with 0.05 T (Fig. 3). The unexpected resistance reduction appears even below the onset of the sudden jump at the critical current, *I*_*C*_, in the *R*_*AC⊥*_.

**Figure 4:**
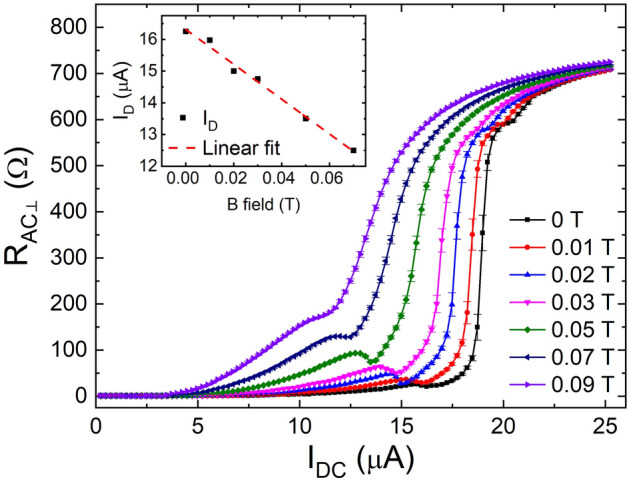
Magnetic field dependence of resistance dip. *I*_*DC*_ dependence of *R*_*AC⊥*_ with various magnetic fields. Inset shows the magnetic field dependence of *I*_*D*_ at which the dip of *R*_*AC⊥*_ appears.

To understand the intriguing reduction systematically, we investigated *I*_*DC*_ dependence of *R*_*AC⊥*_ by increasing magnetic field with a smaller step as shown in Fig. 4. The progressive change of *I*_*DC*_ dependence of *R*_*AC⊥*_ is plotted at various magnetic fields. As the magnetic field increases, the magnitude of dip increases up to 0.05 T and then decreases gradually until it finally disappears at the magnetic field higher than 0.09 T. Although the *R*_*AC⊥*_ dip is not seen at 0.09 T, the slope of *R*_*AC⊥*_ shows clear change, indicating that the surprising effect persists. Further increase of the magnetic field broadens the jump in the *R*_*DC*_ and *R*_*AC⊥*_ and lessens the discrepancy between *R*_*DC*_ and *R*_*AC⊥*_. Non-zero magnetic field reduces *I*_*D*_ with increasing magnetic fields. The *I*_*D*_ shows a linear dependence on the magnetic field as shown in the inset of Fig. 4.

Although it is less realistic, one may explain the enhancement of critical current and the unexpected reduction in *R*_*AC⊥*_ by the enhancement of superconductivity in the superconducting channel between E3 and E7. For example, suppose strong DC current somehow increases the drag force on vortices. Accordingly, reduced are the velocity of the vortex in the flux flow state and the dissipation of vortices in the conduction channel, which can be equivalent to the enhanced superconductivity. The dissipation voltage can be further reduced when the number of vortices present in the superconducting path decreases. However, the escape of vortices to the normal state with increasing DC current can be realized only in the interface so that the additional effect may not be relevant to the further enhancement of the superconductivity. One can also suggest that the proliferation of vortices can form a new spatial order such as a vortex river, alignment of multiple lines of vortices perpendicular to the DC current, in the flux flow state^5,6,9^. The transformation from repulsive vortex-vortex interaction to attractive interaction can lead to the structural distortion of vortex lattice. This new spatial order allows a wider straight conduction channel for *I*_*AC⊥*_ than a complicated path of typical isotropic orders with disorders, which induces a lower voltage drop in the *V*_*AC⊥*_ measurements. We attempted to perform a numerical simulation based on this assumption with the time-dependent Ginzburg–Landau equation, which was previously reported in various sample configurations^9,27,28^. In general, these simulations were conducted on the sample about ten times greater than the superconducting coherence length (~ 10 nm) with further system expansion by the periodic boundary conditions. These simulations provided the vortex dynamics successfully in the samples where uniform current density was assumed with isotropic vortex distribution. However, the vortex dynamics in our square sample was much more complicated because the current density and the vortex distribution are not spatially homogeneous. Accordingly, we could not adopt appropriate periodic boundary condition to allow a reasonable system size for the simulation. This adversity limited our simulation to greatly simplified geometry and current density distribution, which provided only a qualitative explanation of the experimental results. It is notable that the resistance reduction occurs at *I*_*DC*_ where the vortex state shows the flux flow state to the normal state transition. Due to the spatial distribution of current density, the normal state is likely initiated from the high current density region. During the superconducting to normal transition, the superconducting conduction channel of *R*_*AC⊥*_ is expected to become narrower monotonically so that the reduction of *R*_*AC⊥*_ cannot be simply attributed to the spatial current density profile. On the other hand, we found that the progressive increase of *I*_*DC*_ could induce an unusual texture because of the unwanted extra area of the Ta electrodes (E2, E4, E6, and E8) in Fig. 1c. For example, appearance of the normal state in the sample alters a local current density profile dramatically so that the reduction in the current density can be found at the virtual interface between the electrode and the square sample. Further increase of the current leads to the band-like high resistive structure with small low resistive pockets near the electrodes. These interesting transport characteristics were revealed by the model simulation of superconductor-normal transition. (Detailed simulation is described in the "Methods" section.)

**Figure 5:**
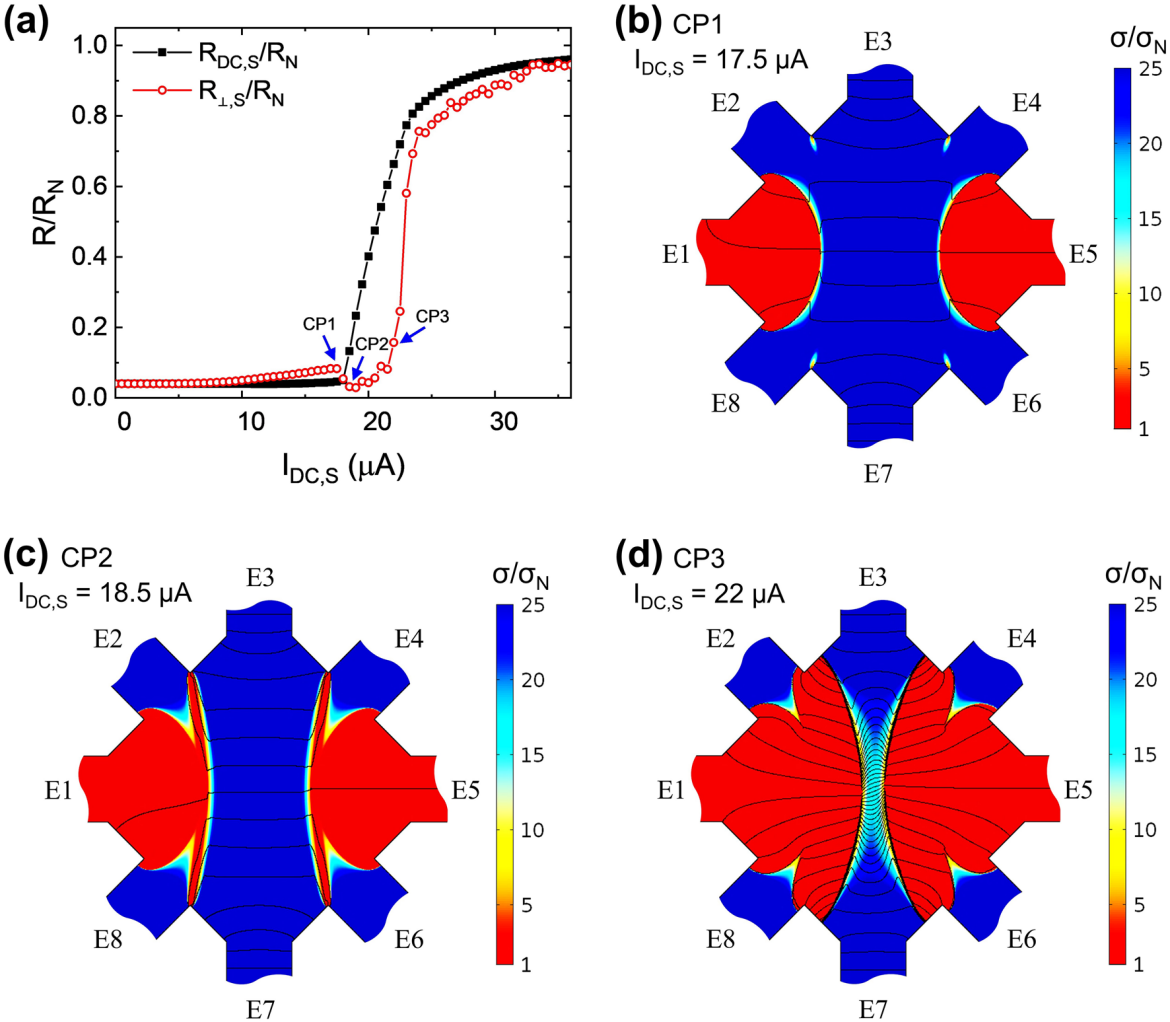
The FEM simulation results. Subscripted S in notations denotes the simulation results that are equivalent to those in the experiments. (**a**) Normalized *R*_*DC,S*_ and *R*_*⊥,S*_ as a function of *I*_*DC,S*_. (**b**)–(**d**) display conductance color maps measured at selected *I*_*DC,S*_ near *R*_*⊥,S*_ dip, marked by blue arrows in (**a**) and labeled as CP1, CP2 and CP3 respectively. Red(blue) indicates low(high) conductance. Black lines show a potential gradient induced by *I*_*⊥,S*_. The potential difference between adjacent lines is all identical in (**b**)–(**d**). The simulation images are generated with COMSOL Multiphysics 5.0 (https://comsol.com).

We displayed the results of the simulation in Fig. 5. Figure 5a shows normalized *R*_*DC,S*_ and *R*_*⊥,S*_ as a function of *I*_*DC,S*_. (Subscripted S in notations indicates “simulation” parameters or variables for easy comparison to those of the experiments.) *R*_*DC,S*_ is obtained by $V_{DC,S} /I_{DC,S}$ where $V_{DC,S} = V_{S} \left( {E2} \right) - V_{S} \left( {E4} \right)$. Similarly, *R*_*⊥,S*_ is obtained by $V_{ \bot ,S} /I_{ \bot ,S}$, where $V_{ \bot ,S} = V_{S} \left( {E2} \right) - V_{S} \left( {E8} \right)$. Both resistances are normalized by the normal state resistance *R*_*N*_ which is calculated with the normal state conductance σ_*N*_ in all the sample. The current dependence of *R*_*DC,S*_ and *R*_*⊥,S*_ essentially reflects the experimental observation shown in Fig. 3. First, *R*_*DC,S*_ exhibits a smaller critical current of 18 μA than that of 21.5 μA in the *R*_*⊥,S*_. About 19% reduction in the simulation is very similar to the experimental results of 15%. Second, the simulation also revealed an intriguing dip structure of *R*_*⊥,S*_ as also found in the experiment. Figure 5b ~ d show a spatial distribution of the conductance induced by *I*_*DC,S*_ at three characteristic points near the distinctive resistance dip. Solid lines in the conductance map show potential distributions induced by solely *I*_*⊥,S*_. The potential difference of two adjacent equipotential lines is selected to be $0.02 \cdot R_{N} \cdot I_{ \bot ,S}$. The red region where the current density is higher than the critical current density is identified as the normal state of which conductance is characterized by σ_*N*_. The blue region is described with conductance 25 times greater than σ_*N*_ and classified into the superconducting state. Conductance distribution profile in Fig. 5b at CP1, just before the rapid change of *R*_*DC,S*_ and *R*_*⊥,S*_, indicates that the superconducting state is well maintained through the conducting paths connecting the voltage probes E2—E4 and E2—E8 respectively. Accordingly, both *R*_*DC,S*_ and *R*_*⊥,S*_ remain small without significant increase. As *I*_*DC,S*_ increases, the normal state grows in the *V*_*DC,S*_ probing path connecting E2 and E4, which can be captured easily in Fig. 5c and d. The emergence of the normal state in the passage hampers the electrical conduction, leading to dramatic increase in *R*_*DC,S*_ as depicted in Fig. 5c. On the other hand, the conduction in the passage connecting the probes E4 and E6 is much more complicated. Suppose the voltage probes (E2, E4, E6, and E8) were absent, monotonic propagation of the normal state toward the center is expected. In the presence of the electrodes, the normal states near E2 (E4) and E8 (E6) progress with a rather sophisticated texture. Because the passage connecting the center of the electrodes E2(E4) and E8(E6) is larger cross section for *I*_*DC,S*_ than that connecting the upper and lower corner of those electrodes, the conductance at the upper corner of E2 (lower corner of E8) is lower than that at the center of E2(E8). Therefore, with increasing *I*_*DC,S*_ the normal state appears in a band-like structure connecting the upper corner of the electrodes E2(E4) and the lower corner of the electrode E8(E6) before the regions near E2(E8) and E4(E6) become the normal state. *R*_*⊥,S*_ shows an unexpected reduction in the short range of *I*_*DC,S*_ from CP1 to CP2 where the high resistive band becomes discernable, which is observed in the experiments(Fig. 4). The reduction can be understood by the fact that the distribution of equipotential lines varies progressively during the evolution. The number of equipotential lines across the sample is increasing with increasing the *I*_*DC,S*_ from CP1 to CP2 because the voltage drops faster in the high resistance region in general. However, the potential difference between E2 and E8 is reduced because of development of the intriguing texture as the simulation progresses from CP1 to CP2. The resistive band lifts up the equipotential line more stiffly compared to the center channel as noted by solid lines in Fig. 5c than that in the conduction via the superconducting passage without the band in Fig. 5b. Accordingly, the measured *V*_*⊥,S*_ with the resistive band can mimic enhanced superconductivity by effectively lowering the potential between two electrodes. It is remarkable that dip structure of *R*_*⊥,S*_ can be eliminated in the comparative simulation where the unusual texture near the voltage probe electrode is minimized by controlling the conductance of electrodes. (See Supplementary Fig. S2.) After CP2, the normal state expands monotonically with increasing *I*_*DC,S*_ and *R*_*⊥,S*_ starts increasing. Finally, the normal states from both ends of E1 and E5 reach the center of the system with further increase of *I*_*DC,S*_ after CP3 and it causes rapid increase of *R*_*⊥,S*._ The simulations show very good agreement with the experiments.

*R*_*DC*_, and *R*_*AC⊥*_ can be anisotropic when the non-steady vortex motion near critical current appears. For instance, the difference in the dissipation mechanism leads to a distinctive DC bias dependence when the anisotropic vortex motion is induced by DC bias. While the anisotropic transport is observed in our experiments, the key features can be understood by the inhomogeneous current density distribution in the sample. We believe this can be the most convincing explanation without introducing the complicated transport mechanism related to the vortex dynamics. However, our measurement cannot strictly confirm whether or not such structural ordering and/or anisotropic vortex motion exist since the unwanted current density profile may mask the trails of the anisotropy induced by the vortex motion.

## Conclusion

We investigated anisotropic transport in the square shape Ta superconducting films. The DC current breaks the isotropic current density profile that introduces intriguing discrepancies in the *R*_*DC*_ and *R*_*AC⊥*_. The critical current can be selectively chosen depending on the measurement direction with about 15% difference. The *R*_*AC⊥*_ exhibits intriguing reduction where the conduction landscape develops very intriguing patterns. Due to the vortex dynamics influenced strongly with the driving force, *R*_*DC*_ and *R*_*AC⊥*_ show anisotropic transport with a finite magnetic field even at low DC bias current. We tried to understand the experimental results with a simple model simulation and found that the intriguing anisotropic transport characteristics could be mostly understood by the inhomogeneous and complex current density profile over the square sample by an unwanted additional geometry of the electrode. Further investigation with a sophisticated design of electrodes is necessary to confirm the underlying mechanism for anisotropic transport and intrinsic vortex dynamics.

## Methods

### Sample fabrication and transport measurement

Ta thin films with various thicknesses were fabricated by a DC sputtering technique on a SiO_2_/Si substrate. The fabrication method and characteristics of the films are the same as those reported previously^18^. The films were patterned into a square with 8 electrodes for an anisotropy measurement with a shadow mask (see Fig. 1a). All the samples showed essentially the same conduction characteristics, and the data presented in the article are obtained from the Ta film of a thickness of 4 nm. Additional transport data of Ta thin films with thicknesses of 3.4 and 3.7 nm are included in the Supplementary Information (see Fig. S1). *R*_*DC*_, *R*_*AC⊥*_ and *R*_*AC∥*_ were measured with a source-meter and two lock-in amplifiers. The source-meter was used for applying *I*_*DC*_ and measuring *V*_*DC*_. The *R*_*AC∥*_ was obtained by a lock-in amplifier while *I*_*AC∥*_ of 7.77 Hz was superposed to *I*_*DC*_ by using a home-made OP-amp circuit. The *R*_*AC⊥*_, on the other hand, was detected using another lock-in amplifier, while *I*_*AC⊥*_ of 13.55 Hz is floating with respect to a common ground using a transformer. Both *I*_*AC∥*_ and *I*_*AC⊥*_ were fixed at 100 nA, which was great enough to allow continuous *V*_*AC∥*_ and *V*_*AC⊥*_ detection without disturbing DC measurements. Low-temperature measurements are performed with a home-made cryo-free dilution refrigerator with a superconducting magnet. The magnetic fields are applied perpendicular to the sample plane.

### Estimation of the critical current density

The exact determination of the critical current density is not trivial due to the inhomogeneous current density distribution in the square geometry. Thus, we estimate the critical density at the center of the sample in two different ways. First, we assume the current propagates like a circular wave from a source point at the corner. Then, the area of a cross-section which maintains the identical current density is estimated by multiplying the arc length of a quarter circle of which the radius is half of the sample diagonal(= $\sqrt 2$ mm) with the sample thickness(= 4 nm). This shape of the quarter circle is similar to the normal region in Fig. 5d. The critical current for the center is determined from the abrupt change of *R*_*AC⊥*_ in Fig. 3, which is 18.75 μA. From the measured critical current and estimated cross-section area, we obtain the critical current density of 2.13 MA/m^2^. Second, the critical current density can be also estimated by the simulation in Fig. 1c. Since the exact area of the cross-section with the identical current density cannot be directly measured, one may utilize the simulation to estimate the ratio of the current density at the center to the known current density at the current probes (E1 and E5). The current density at the current probes is the applied current divided by its cross-section area, i.e., the width of the electrode(= 0.5 mm) times the sample thickness(= 4 nm). Because the current density of the center is 3.51 times lower than that at the electrode, we obtain the critical current density of the center, 2.67 MA/m^2^ at the applied current of 18.75 μA. The values of the critical current density determined by two difference methods are comparable and in a good agreement with the previous results ^12,18^.

### FEM simulation of current density profile

**Figure 6:**
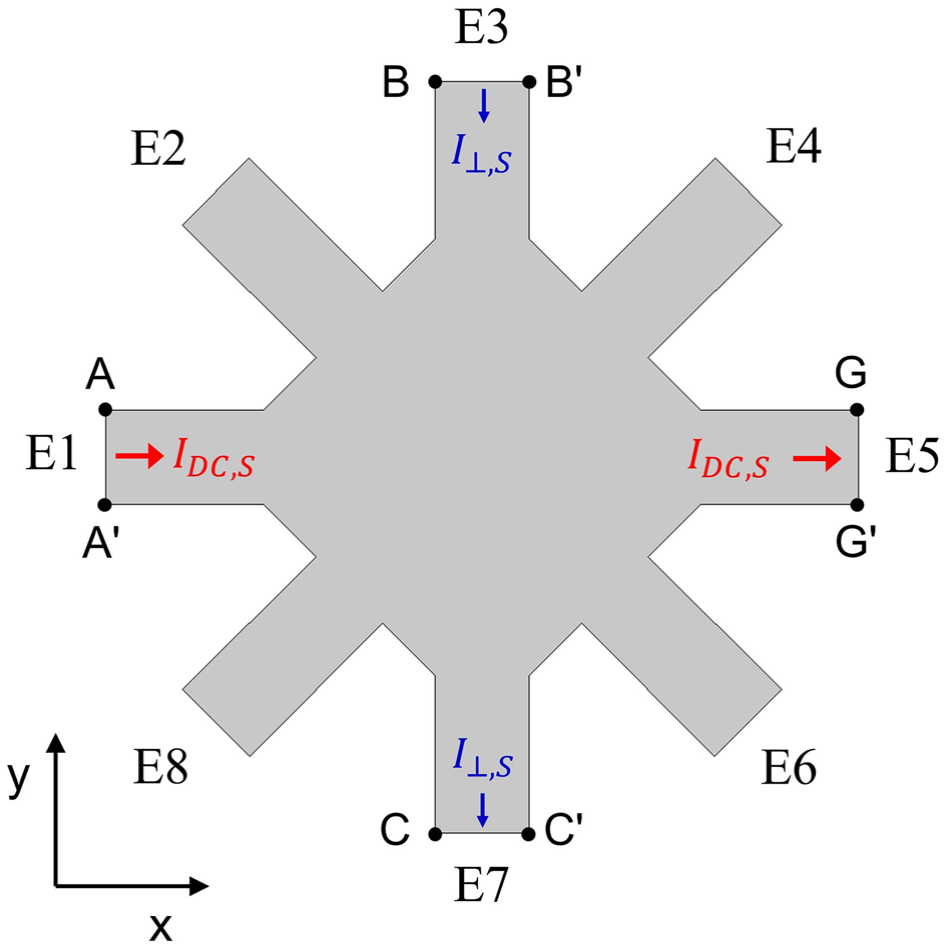
Schematic diagram of the FEM simulation geometry. The gray area represents simulation region. A-A’, B-B’, C–C’ and G-G’ boundaries have specific boundary conditions given by the applied current, which is described in Eq. (1). All other electrodes are electrically isolated.

The voltage distribution (*V*_*S*_) in the sample geometry was calculated with FEM simulation. The governing equations in the simulation is that the charge conservation (current continuity) equation which is equivalent to the Maxwell’s equation, $\nabla \cdot {\varvec{J}} = - \partial \rho /\partial t$, and the Ohm’s law, ${\varvec{J}} = \sigma {\varvec{E}}$ where ***J*** is current density, *ρ* is charge density, σ is conductance and ***E*** is electrical field. The system geometry of the simulation is selected to be identical to the sample geometry used in the experiment, shown in the Fig. 6. It has a square shape of 2 mm length on each side and identical electrodes with 0.5 mm width placed at all vertices and the center of each side. The film of thickness 4 nm is assumed to be nearly two dimensional. The current flow can be induced with the boundary conditions described in the Fig. 6.

$$\mathop \int \limits_{{\partial \Omega \left( {A - A^{\prime}} \right)}}^{{}} \hat{x} \cdot \varvec{J}dS = \mathop \int \limits_{{\partial \Omega \left( {G - G^{\prime}} \right)}}^{{}} \hat{x} \cdot \varvec{J}dS = I_{DC,S}$$

1 $$\mathop \int \limits_{{\partial \Omega \left( {B - B^{\prime}} \right)}}^{{}} - \hat{y} \cdot \varvec{J}dS = \mathop \int \limits_{{\partial \Omega \left( {C - C^{\prime}} \right)}}^{{}} - \hat{y} \cdot \varvec{J}dS = I_{ \bot , S}$$

These boundary conditions indicate that the injection of current *I*_*DC,S*_(*I*_*⊥,S*_) through the A-A’(B-B’) boundary is the same as the ejection of current through the G-G’(C–C’) boundary. This simulation aims to reproduce the experimental set-up in the same way so that the current flow can be ideally identical to that of the real experiment. We constructed finite elements with free triangle meshes size in 40 nm to 10 μm and conducted the simulation with the FEM module in COMSOL multiphysics. Figure 1c is the simulation result with *I*_*DC,S*_ = 1 μA, *I*_*⊥,S*_ = 0 and σ = 1 S/m.

### FEM simulation with special conductance condition

As discussed in the Results and discussion section, our experimental setup leads to the non-uniform current density distribution in the sample. We assumed that the conductance, σ, exhibits dramatic change in the spatial profile near the critical current density accordingly. Because of the superconductor-normal(SN) transition at critical current density, presumably the conductance can be estimated by simple combination of the normal state conductance, σ_*N*_, and the superconducting conductance of σ_*S*_. Then, the current dependence of the sample conductance can be given by

2 $$\sigma \left( J \right) = \left\{ {\begin{array}{*{20}c} {\sigma _{S} ,\quad J < J_{C} } \\ {\sigma _{S} - \frac{{\sigma _{S} - \sigma _{N} }}{\alpha } \cdot \left( {J - J_{C} } \right),\quad J_{C} \le J < J_{C} + \alpha } \\ {\sigma _{N} ,\quad J \ge J_{C} + \alpha } \\ \end{array} } \right.$$

where *J*_*C*_ is critical current density, 2.5 MA/m^2^, and the adjusting parameter *α* is introduced for avoiding discontinuity of which value is 50 times smaller than *J*_*C*_. Although the conductance of superconducting state should be infinite, superconducting state we concern here is in flux flow state so that it has finite value. We assumed the ratio of superconducting to normal state conductance is 25 and its value is sufficient for capturing the intriguing conductance change during the SN transition in the simulation. In the simulation, *I*_*DC,S*_ is increased from 0 to 40 μA with 0.5 μA step while *I*_*⊥,S*_ is fixed at 100 nA, which is negligible in changing the conductance. We obtained the essentially identical simulation results with manipulating functional forms of linear, cubic, and reciprocal dependence of $\sigma \left( J \right)$, changing *α* in the range of 0.5 to 50% of *J*_*C*_, and altering the ratio of the conductance (σ_*S*_/σ_*N*_) in the range of 5 to 100. The main features reproduced consistently in these simulations and were not affected by shifting the critical current density value.

## Supplementary information

Supplementary information.

## Data Availability

The datasets generated or analysed during the current study are available from the corresponding author on reasonable request.
