# Supplementary material for: Anisotropic transport induced by DC electrical current bias near the critical current
Source: Sci Rep. 2020 Oct 8;10:16841. doi: 10.1038/s41598-020-73876-6 (PMC7546633; doi:10.1038/s41598-020-73876-6)
Supplement: Supplementary file 1 — Supplementary information. [file 41598_2020_73876_MOESM1_ESM.pdf]

Supplementary Information
: Anisotropic transport induced by DC electrical current bias near the critical current

Junghyun Shin[1], Sungyu Park[1,2*] and Eunseong Kim[1]

[1]Department of Physics, KAIST, Daejeon, 34141, Republic of Korea.

[2]Present address: Center for Artificial Low Dimensional Electronic Systems, Institute for Basic Science (IBS), Pohang 37673, Republic of Korea

[*] Corresponding author(e-mail : tour424@kaist.ac.kr)

This supplementary information includes:

- DC bias dependence of R_{DC} and $R_{AC\perp}$ in Ta thin films with various thickness
  - Supplementary Figure S1
- FEM simulation with controlled conductance of the electrodes
  - Supplementary Figure S2

DC bias dependence of R_{DC} and $R_{AC\perp}$ in Ta thin films with various thickness.

Transport measurements of R_{DC} and $R_{AC\perp}$, which are identical to those plotted in Fig. 3 in the main article, are shown in Fig. S1 (a) for the sample #2 and (b) for #3. The film thickness of the sample #2 (#3) is 3.7 (3.4) nm and the normal resistance at T = 1 K is 952 (1029) Ω with T_C = 360 (310) mK and B_C = 0.50 (0.48) T. We found the qualitatively equivalent transport properties with that of the sample in main text such as the shift in the critical currents between R_{DC} and $R_{AC\perp}$ and the unexpected resistance reduction of $R_{AC\perp}$.

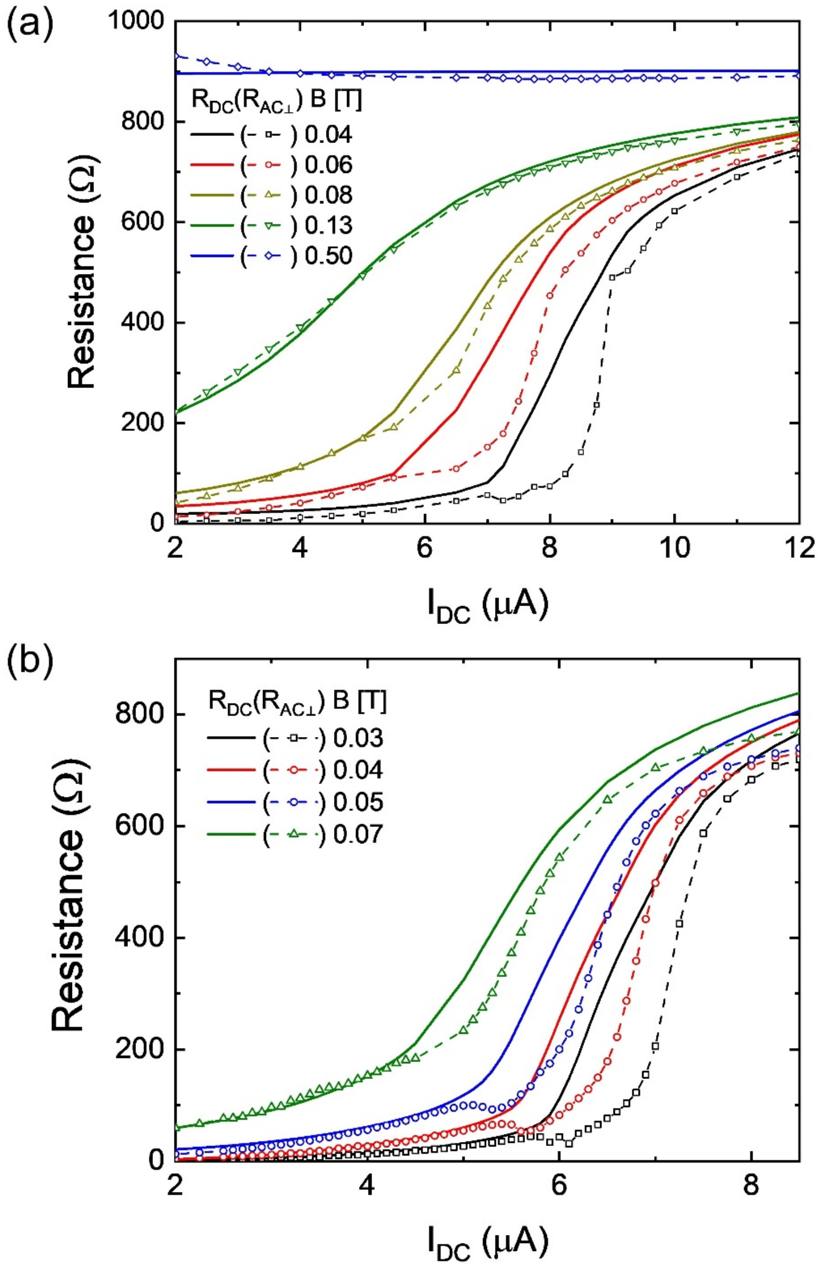

Supplementary Figure S1. Simultaneous measurement of R_{DC} and $R_{AC\perp}$. I_{DC} dependence of R_{DC} (straight lines) and $R_{AC\perp}$ (open symbols with dashed lines) perpendicular to I_{DC} with various magnetic fields in (**a**) Sample #2 and (**b**) Sample #3

FEM simulation with controlled conductance of the electrodes

We conducted a control simulation to validate the explanation for the drop in the $R_{\perp,S}$ in the main text. We utilized the identical simulation setup and method described in the main text with a small modification to achieve separate conductance control for the electrodes, which is crucial for verifying the effect of an electrode on a current density distribution. The system was divided into two parts (eight electrodes and a center square) as shown in the inset of Fig. S2(a). The conductance of two parts was set to have different values. The conductance $\sigma(J)$ described in the Method section is assumed for the square part (gray color). On the other hand, the constant conductance σ_E for each electrode (green color) with three selected values of σ_N, $0.1\sigma_N$, and $0.01\sigma_N$ were assigned to test the electrode effects. The system becomes analogous to the sample without electrode for $\sigma_E = 0.01\sigma_N$ as shown in Fig. S2(b), which demonstrates that these electrodes with small conductance minimize the inhomogeneity in the current density (See the main text).

Figure S2(a) shows the simulation result of the normalized $R_{\perp,S}$ as a function of $I_{DC,S}$ with various σ_E. As σ_E decreases, the reduction becomes weaker and, finally, no reduction is observed at $\sigma_E = 0.01\sigma_N$ (no electrode limit). Figure S2(b) shows the conductance map induced by $I_{DC,S} = 18.5$ μA [the same current level as that in Fig. 5(c)] and equipotential line distributions induced by $I_{\perp,S}$. The resistive band structure connecting the upper corner of E4 and the lower corner of E6 disappears in striking contrast to that of the original simulation in the main article [Fig. 5(c)]. Accordingly, this control simulation justifies the dominant role of the additional voltage probes (electrodes E2, E4, E6, and E8) for the unexpected transport characteristics.

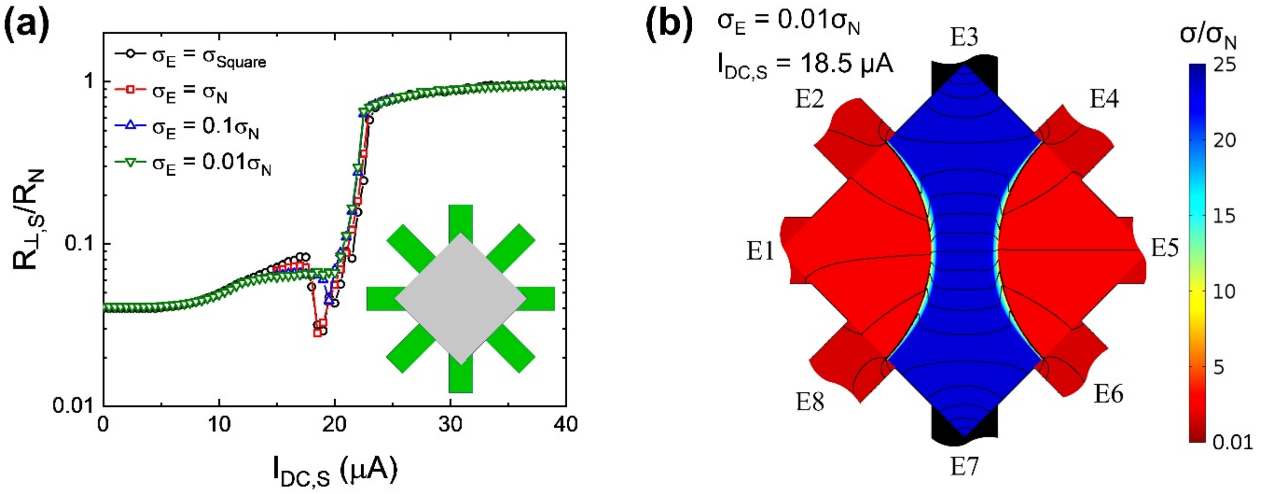

Supplementary Figure S2. FEM simulation with controlled σ_E. (**a**) Perpendicular resistance $R_{\perp,S}$ as a function of $I_{DC,S}$ with various electrode conductance σ_E. The inset shows a schematic diagram of the simulation geometry with two separated parts (a square in gray and eight electrodes in green). σ_{Square} represents the conductance of a square. (**b**) The conductance color maps at $I_{DC,S} = 18.5$ μA with $\sigma_E = 0.01\sigma_N$. (The simulation image is generated with COMSOL Multiphysics 5.0. http://comsol.com)
